# Isolated fallopian tube torsion associated with hydrosalpinx in a 12-year-old girl: a case report

**DOI:** 10.1186/s13256-020-02462-1

**Published:** 2020-09-14

**Authors:** Javier Martín-Vallejo, Enrique E. Garrigós-Llabata, Patricia Molina-Bellido, Pedro A. Clemente-Pérez

**Affiliations:** Department of Obstetrics and Gynecology, Hospital de Denia, Avenida Marina Alta, s/n, 03700 Denia, Alicante Spain

**Keywords:** Case report, Conservative management, Fertility, Hydrosalpinx, Isolated fallopian tube torsion

## Abstract

**Background:**

Isolated fallopian tube torsion associated with hydrosalpinx is a rare condition in the pediatric population. We present this unusual clinical case study in a sexually inactive girl.

**Case presentation:**

a12-year-old Caucasian girl presented symptoms of acute abdominal pain. Pelvic ultrasound revealed a normal looking uterus and ovaries and next to left ovary a imaging compatible with hydrosalpinx. She was discharged 48 hours later after clinical monitoring with oral analgesia and normal blood workup. At 3 weeks, she was readmitted for acute abdominal pain. Leukocytosis with left shift and raised C-reactive protein were observed. Her clinical condition worsened, and complication of the preexisting hydrosalpinx was suspected. Exploratory laparoscopy confirmed torsion of the fallopian tube. Left salpingectomy was performed. Histopathologic study confirmed a fallopian tube with hemorrhagic infarct.

**Conclusion:**

Torsion of the fallopian tube must be considered in the event of acute abdominal pain. Early diagnosis and trying conservative management with a view to preserving fertility in this group of patients are essential.

## Introduction

Several intrinsic or extrinsic predisposing factors have been identified in adults as being associated with isolated fallopian tube torsion (IFTT) [1]. However, in sexually inactive girls or adolescents, a possible factor is preexisting congenital malformations such as hydrosalpinx [2].

Hydrosalpinx can occur free of symptoms; however, when associated with IFTT, it is usually accompanied by symptoms of nonspecific abdominal pain [2]. This condition is hard to diagnose with standard imaging tests, and surgical examination is needed to make a final diagnosis [3]. Surgical treatment of IFTT is controversial [1] and ranges from a wait-and-see approach to total salpingectomy. Early diagnosis is essential to consideration of conservative management [3].

## Case presentation

A 12-year-old Caucasian girl came to the emergency department of our hospital. She reported abdominal pain of 48-hour clinical course located in the left iliac fossa as well as feeling nauseous. The patient reported similar clinical symptoms, which were self-limiting, the month prior. She did not present with any medical or surgical history of note, and her gynecological history revealed that she had not undergone menarche and had not yet commenced sexual relations.

**Fig. 1:**
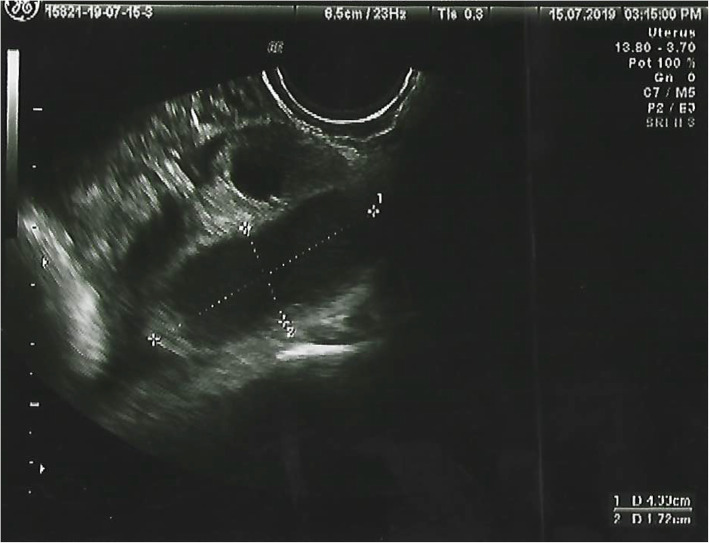
Pelvic ultrasound showing hydrosalpinx and normal left ovary

**Fig. 2:**
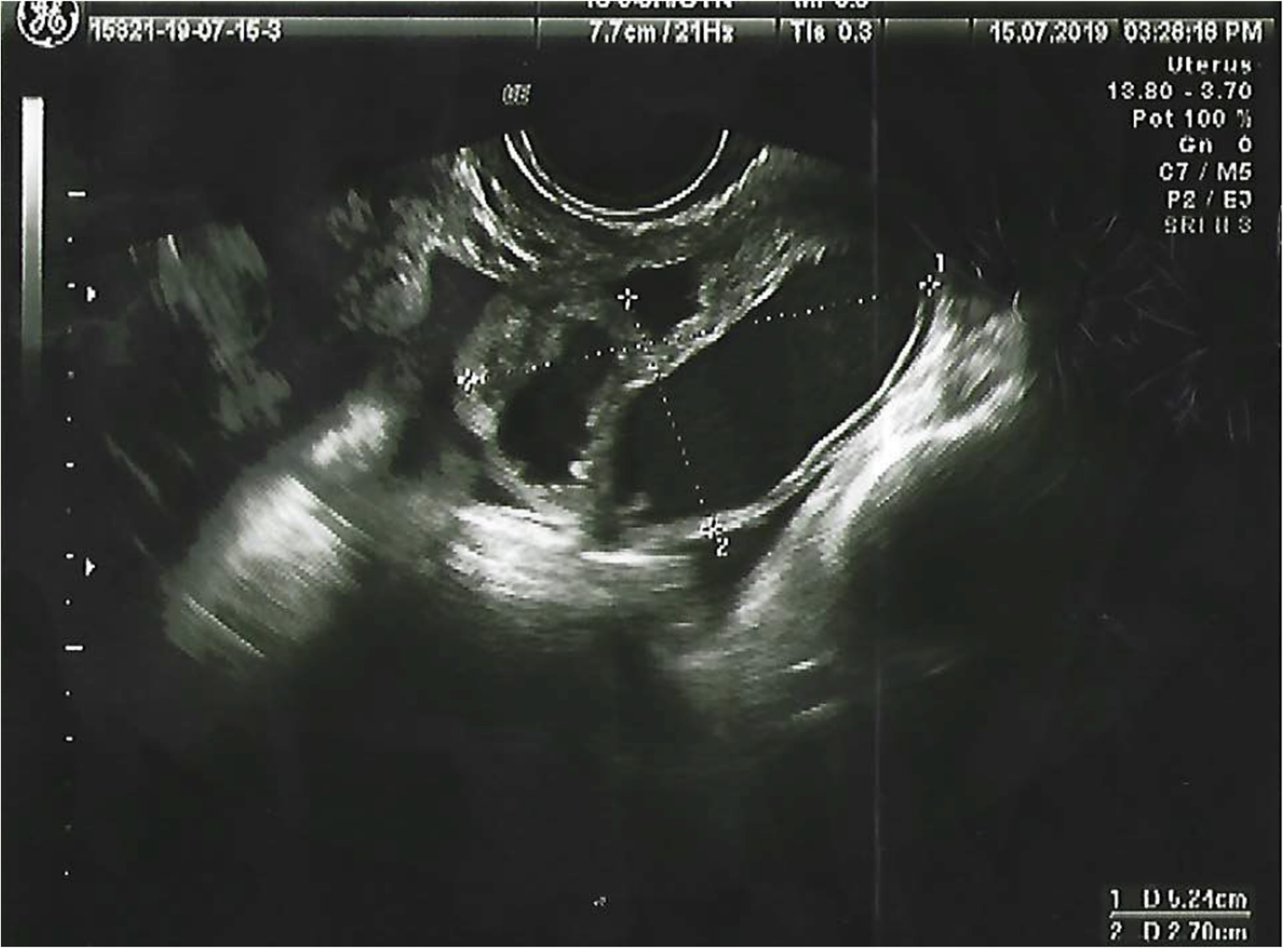
Pelvic ultrasound showing hydrosalpinx

Her physical examination revealed normal vital signs and no fever. We observed normal development of secondary sexual characteristics in Tanner stage 3–4. She presented with abdominal pain on deep palpation in the left iliac fossa, was free of signs of peritoneal irritation, and had no organ masses or organomegaly. Additional tests revealed mild leukocytosis (11.1 × 10^3^/μl) with left shift (81.2% neutrophils) and negative C-reactive protein (CRP; < 0.5 mg/L). The results of basic analysis of normal urine and pregnancy tests were negative. We performed abdominal and transrectal ultrasound and visualized the patient’s uterus and ovaries of normal size, form, and echogenicity. Adjacent to the left ovary, an elongated formation of approximately 52 × 27 mm was seen, with anechoic content and negative color Doppler map, which suggested hydrosalpinx. No free fluid in the pouch of Douglas was observed (Figs. 1 and 2).

Given the above findings, hospital admission and observation were decided. Oral analgesia with 650 mg of paracetamol every 8 hours was prescribed. After 48 hours, the patient’s symptom of abdominal pain receded. No leukocytosis with a negative CRP was observed. Given the stability of the clinical symptoms, and having ruled out the complication of hydrosalpinx, hospital admission and monthly monitoring in gynecology consultation were decided.

**Fig. 3:**
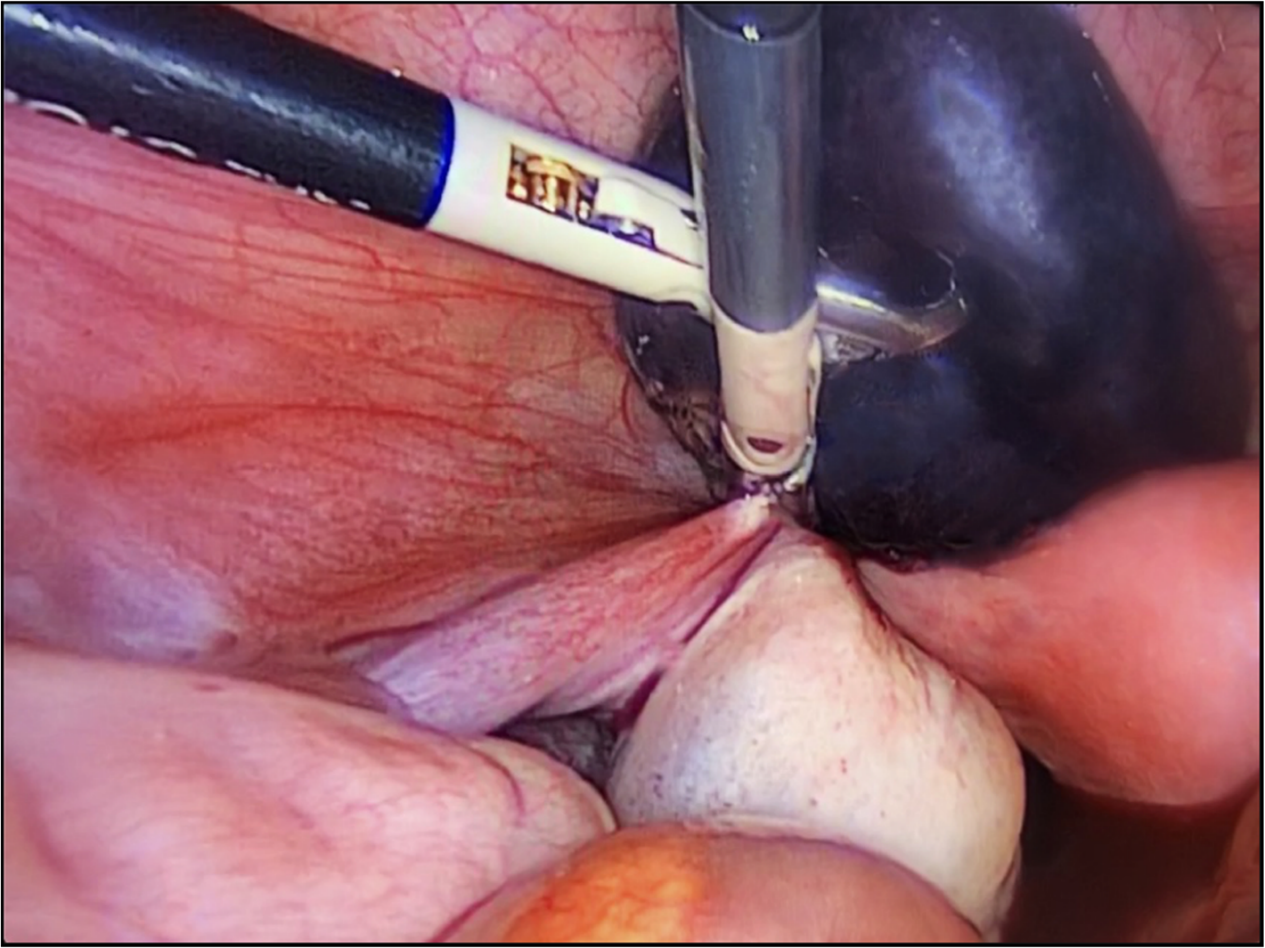
Laparoscopic view of isolated Fallopian tube torsion

After 3 weeks, the patient was readmitted because of abdominal pain, nausea, and vomiting of several hours’ clinical course. She had presented menarche the week prior to readmission. Upon physical examination, she presented with intense pain in the left iliac fossa with signs of peritoneal irritation. Ultrasound revealed the same left para-adnexal formation and existence of a moderate amount of free fluid in the pouch of Douglas. Her blood workup had worsened, with leukocytosis (13.5 × 10^3^/μl), left shift (83.9% neutrophils), and elevated CRP (24 mg/L). Given these findings and suspected complications, exploratory laparoscopy was indicated. During the intervention, we observed that the left fallopian tube was increased in size, with torsion on its own axis, signs suggesting ischemia and impossible detorsion. We proceeded to perform left salpingectomy (Fig. 3). On a macroscopic level, subsequent pathologic study of the surgical specimen revealed a violet-colored tube measuring 53 × 35 × 20 mm. Microscopically, the existence of tissue with hemorrhagic infarction free of evidence of malignancy was revealed.

## Summary and conclusion

IFTT is a rare finding in pediatric patients, marked by its rotation on its own axis without involving the ipsilateral ovary. Bertozzi *et al.* [4] reported just 20 cases in 26 years at five pediatric surgery units. In the same way, it is a rare cause of abdominal pain in this age group and is exceptional in premenarchal girls [2]. Accompanying symptoms are very nonspecific, such as nausea and vomiting [3]. As for laterality, adnexal torsion is more common on the right side because the sigmoid colon can act as an obstacle on the left side [5].

Risk factors associated with IFTT include endometriosis, adhesion-related disorder, and tubal pathology, such as hydrosalpinx, hematosalpinx, neoplasm, or congenital malformation [3], with hydrosalpinx being the most frequent cause [4]. Some studies suggest that some sports that involve sudden body movements could precipitate torsion of the fallopian tube [5].

In regard to further tests, blood analysis should be performed to guide the initial diagnostic suspicion. Nonetheless, both leukocytosis and raised CRP are not specific to this condition. Ultrasound should be the first imaging test to perform because of its availability and diagnostic yield. The existence of a normal ovary with adjacent imaging of cystic appearance is useful for making a preoperative diagnosis of IFTT. Biology (tumor markers, human chorionic gonadotropin for extrauterine pregnancy) may be useful according to personal history and ultrasound findings. Magnetic resonance imaging is a valid alternative but is less accessible and less efficient [6]. However, surgical exploration is the only way to confirm the clinical suspicion of IFTT. Exploratory laparoscopy is used as an initial surgical and diagnostic option in the vast majority of cases [4, 7].

Ideally, conservative surgery is attempted, opting for detorsion of the fallopian tube with a view to preserving the patient’s fertility, although this option can vary according to the surgeon’s choice [6]. Clinical follow-up and ultrasound study until adult age are necessary. Various pathologic studies have revealed that, although the tube’s microscopic appearance looks necrotic, microscopic study can provide information on integrity in hair cells [2, 7]. Salpingectomy is controversial because of current recommendations in regard to cases of ovarian torsion. Even in ovaries revealed to have a necrotic appearance, it is recommended to perform conservative surgery by means of detorsion of the annex without this having been demonstrated to increase morbidity [8]. In cases of isolated hydrosalpinx free of symptoms and with no complications, a wait-and-see approach can be considered because cases of spontaneous resolution have been reported [7].

If, during exploratory laparoscopy, IFTT is confirmed, the surgeon should opt for conservative management or perform salpingectomy according to macroscopic characteristics, possibility of detorsion and signs of revascularization, in accordance with the algorithm proposed in a multicenter study [4]. In our patient’s case, we were unable to perform conservative management, given that detorsion of the left fallopian tube was impossible; we had to perform a salpingectomy to resolve the clinical symptoms.

To conclude, IFTT has a nonspecific clinical presentation, and preoperative suspicions are rare. Pelvic abdominal pain must be considered as a differential diagnosis in children, especially if ultrasound study reveals a pelvic tubular structure without involvement of the ovary.

Due to its rarity, case reports and small studies are the only source of information available in the medical literature. A long-term prospective study would be needed to determine whether future fertility is affected in cases of detorsion and revascularization of the fallopian tube.
